# Isolation of cell-free bacterial inclusion bodies

**DOI:** 10.1186/1475-2859-9-71

**Published:** 2010-09-17

**Authors:** Escarlata Rodríguez-Carmona, Olivia Cano-Garrido, Joaquin Seras-Franzoso, Antonio Villaverde, Elena García-Fruitós

**Affiliations:** 1 Institut de Biotecnologia i de Biomedicina and Departament de Genètica i de Microbiologia, Universitat Autònoma de Barcelona, 08193 Bellaterra (Cerdanyola del Vallès), Barcelona, Spain; 2 CIBER en Bioingeniería, Biomateriales y Nanomedicina (CIBER-BBN), Bellaterra, 08193 Barcelona, Spain

## Abstract

**Background:**

Bacterial inclusion bodies are submicron protein clusters usually found in recombinant bacteria that have been traditionally considered as undesirable products from protein production processes. However, being fully biocompatible, they have been recently characterized as nanoparticulate inert materials useful as scaffolds for tissue engineering, with potentially wider applicability in biomedicine and material sciences. Current protocols for inclusion body isolation from *Escherichia coli *usually offer between 95 to 99% of protein recovery, what in practical terms, might imply extensive bacterial cell contamination, not compatible with the use of inclusion bodies in biological interfaces.

**Results:**

Using an appropriate combination of chemical and mechanical cell disruption methods we have established a convenient procedure for the recovery of bacterial inclusion bodies with undetectable levels of viable cell contamination, below 10^-1 ^cfu/ml, keeping the particulate organization of these aggregates regarding size and protein folding features.

**Conclusions:**

The application of the developed protocol allows obtaining bacterial free inclusion bodies suitable for use in mammalian cell cultures and other biological interfaces.

## Background

Bacterial inclusion bodies (IBs) are water-insoluble protein aggregates formed in the bacterial cytoplasm (and eventually periplasm) during the overproduction of recombinant proteins, especially those from viral or mammalian origin [1]. The diameter of these insoluble proteinaceous particles range from about 50 nm to 500 nm, depending on the background of the producer strain, harvesting time, culture conditions and recombinant protein [2]. Although IBs have traditionally been described as biologically inert protein clusters, recent insights show that these nanoparticles have not only an important level of molecular organization, but also that they are formed by a considerable extent of functional polypeptides [3-7]. In fact, IBs are pure [8], structurally organized [9], mechanically stable and biocompatible protein deposits [2], formed through stereospecific protein-protein cross-molecular interactions under amyloid-like schemes [9,10]. This turn in the understanding of IB biological nature has prompted to explore potential applications of such aggregates as straightforward bacterial products. One of the major IB applications is their use as particulate catalysts for different bioprocesses when formed by enzymes. It has been successfully proved with different enzyme-based IBs that these aggregates efficiently catalyse bioprocesses, becoming a promising alternative to classical enzyme immobilization [7,11]. In the completely different context of tissue engineering and regenerative medicine, it is widely accepted that the nano- and micro-modification of flat surfaces by different procedures, such as etching, lithography and particle decoration, can not only favour mammalian cell binding but also improve cell proliferation and substrate colonization [12]. In this regard, we have recently explored the performance of IBs as biocompatible particulate materials suitable for engineering surfaces roughness at a micro- and nano-scale level to stimulate, by mechano-transduction events, the growth of cultured mammalian cells [2,13,14].

On the other hand, a wide range of protocols for the purification of cytoplasmic IBs are available, all of them including bacterial lysis and IB washing steps. Bacterial lysis is achieved using either mechanical or non mechanical methods, or a combination of both, while washing steps include, among others, detergent and/or DNase treatments. These protocols have been mainly aimed to obtain IBs suitable for in vitro protein refolding attempts [15]. Since most of them permit the recovery of high amounts of pure IBs (usually representing between 90 and 95% of the total aggregated proteins), the presence of viable bacteria in the final sample is not routinely verified. Residual bacterial contamination may not be a critical issue when using IBs as the starting material for protein refolding procedures [16-18] (Table 1[19-58]). However, for applications in which IBs act as materials in biological interfaces, for instance in tissue engineering [2] or as biocatalysts [11], the presence of living bacteria would be not acceptable.

**Table 1:** Bacterial lysis methods for IB purification.

Lysis method		IB protein	DNase	Detergents	Tested viability	Reference
**NON MECHANICAL LYSIS METHODS**	**LYSOZYME**	LACVP1	Yes	Yes	No	[19-22]
		
		VP1LAC	Yes	Yes	No	[7-9,19-27]
		
		V2LAC	Yes	Yes	No	[22]
		
		TSP	Yes	Yes	No	[23]
		
		VP1GFP	Yes	Yes	No	[7,28-30]
		
		hDHFR	Yes	Yes	No	[7]
		
		Aβ42-BFP	Yes	Yes	No	[7]
		
		β-lactamase	Yes	Yes	No	[31]
		
		Prochymosin	No	Yes	No	[32]
		
		HET-s fungal prion	Yes	Yes	No	[33]
		
		Aβ42-GFP	Yes	Yes	No	[30,34]
		
		Aβ42-BFP	Yes	Yes	No	[30]
		
		MalE-Bla and MalE31-Bla	No	No	No	[35]
		
		MalE-PhoA and MalE31-PhoA	No	No	No	[35]
	
	**NON-IONIC DETERGENTS**	CBDclosN-SAA	No	Yes	No	[36]
		
		SAA-6HisC	No	Yes	No	[36]
		
		Maltodextrin phosphorylase	No	Yes	No	[37]
		
		CBD_clos_SabA	No	Yes	No	[38]

**MECHANICAL LYSIS METHODS**	**HOMOGENIZER**	rhBMP-2	No	Yes	No	[17,39]
		
		G-CSF	No	No	No	[40]
		
		rhMCSF	No	No	No	[41]
		
		rHEWL	No	Yes	No	[42]
		
		GFP	No	No	No	[43]
	
	**FRENCH PRESS**	EGD	No	No	No	[44]
		
		TvDAO	No	Yes	No	[45,46]
		
		NS3 protein	No	Yes	No	[47]
	
	**SONICATION**	IFN-α	No	No	No	[48]
		
		β-galactosidase	No	No	No	[49]
		
		pGH	No	No	No	[50]
		
		Pre-β-lactamase	No	No	No	[51]
		
		Procathepsin B	No	Yes	No	[52]

**COMBINED LYSIS METHODS**	**SONICATION** **+** **HOMOGENIZER**	N^pro ^fusion proteins	No	Yes	No	[53]
	
	**SONICATION** **+** **LYSOZYME** **or** **LYSOZYME** **+** **SONICATION**	Prochymosin	No	Yes	Yes	[54]
		
		Cro-β-gal	Yes	Yes	Yes	[55]
		
		His-GST-GFP	Yes	No	No	[56]
		
		Prochymosin B	No	Yes	No	[54]
		
		CLIPB14 Serine protease	Yes	Yes	No	[57]
	
	**FRENCH PRESS** **+** **LYSOZYME** **or** **LYSOZYME** **+** **FRENCH PRESS**	PHA synthase	Yes	Yes	No	[58]
		
		Class II PHA synthase	Yes	Yes	No	[58]

The aim of this study has been the development of an IB purification protocol rendering bacterial-free protein particles, which could not compromise the applicability of IBs as biomaterials for biomedical applications.

## Results

**Figure 1:**
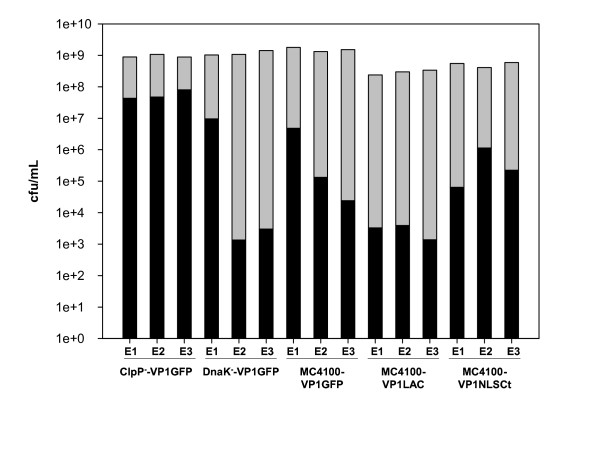
**Viable cells counts before (grey bars) and after (black bars) cell lysis, using a standard protocol based on lysozyme and detergent treatment**. E1, E2 and E3 correspond to three different replicas.

**Figure 2:**
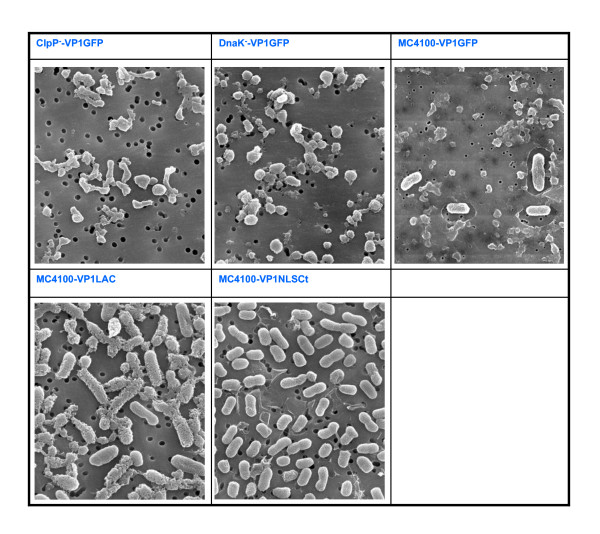
**Scanning Electron Microscopy (SEM) images of MC4100, DnaK^- ^and ClpP^- ^overexpressing VP1GFP (top) and of MC4100 overexpressing VP1LAC and VP1NLSCt (bottom)**.

To explore the efficiency of conventional IB purification protocols based on lysozyme treatment combined with repeated detergent washing steps, we have determined the number of viable bacterial cells before and after cell lysis, using different bacterial strains (MC4100, DnaK^- ^and ClpP^-^) carrying plasmids encoding different recombinant proteins (VP1GFP, VP1LAC and VP1NLSCt) (Figure 1). Our results indicate that the used standard protocol is inefficient concerning the complete removal of viable bacteria (Figure 1). The integrity of bacterial cells upon IB purification was confirmed by scanning electron microscopy (SEM) (Figure 2). Comparing the results obtained with *Escherichia coli *MC4100 carrying different plasmids, we noticed that, being the initial amount of bacteria around 1·10^9 ^cfu/ml in all cases, remaining viable cells after the protocol application ranged from 5·10^7 ^to 1·10^3 ^cfu/ml (Figure 1). In particular, we noted that the ClpP^- ^strain carrying pTVP1GFP plasmid was the most resistant to cell lysis, since more than 10^7 ^cfu/ml remained after IB purification (Figure 1). Intriguingly, replicas of ClpP^-^/pTVP1GFP and MC4100/pTVP1LAC cultures gave quite similar viable cell counts, but the lysis of DnaK^-^/pTVP1GFP, MC4100/pTVP1GFP and MC4100/pTVP1NLSCt showed an important variability (Figure 1).

**Figure 3:**
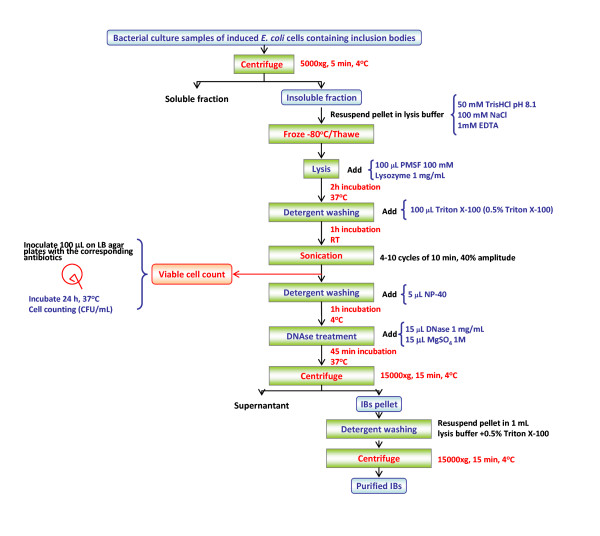
**IB purification protocol: lysozyme-detergent, sonication and repeated detergent washing treatment**.

**Figure 4:**
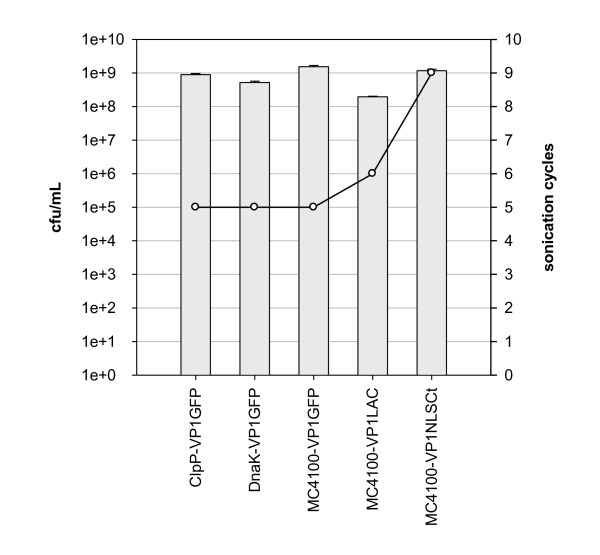
**Viable cells counts in initial bacterial cultures (grey bars) and sonication cycles needed to eliminate all viable bacteria (white circles)**.

Because of the poor performance of the standard protocol cell lysis based on lysozyme, we decided to explore the effectiveness of other cell disruption protocols (Table 1). Viable bacteria were still observed after the application of lysis methods such as the French Press and freeze-thawing (data not shown). Specifically, we determined the viable cell concentration after using the French Press up to 7 rounds at 2,000 psi, observing a non significant decrease in the viable cell counts. Additionally, the effectiveness of freeze-thawing rounds was also not relevant. Thus, neither the physical nor the chemical methods tested were effective enough to obtain IBs free from bacterial contamination. Therefore, as the bacterial lysis was a bottleneck of the whole IB purification process, we decided to develop an improved protocol by combining both sonication and lysozyme treatment. After testing several combinations of these procedures and determining cell counts at the end of each process, the best protocol (Figure 3) combined both physical and chemical lysis methods with some washing steps and a DNase treatment (Figure 3). After the sonication step (Figure 3), no viable bacteria were observed in the sample containing purified IBs, regardless of the strain and plasmid used. However, the number of sonication cycles needed to eliminate all viable bacteria clearly depended on the particular protein encoded in the plasmid (Figure 4). Full lysis of *Escherichia coli *strains overproducing VP1GFP needed 5 sonication rounds of 10 min at 40% amplitude under 0.5 s cycles, independently of the genetic background (Figure 4). However, strains overproducing VP1LAC or VP1NLSCt proteins required 6 and 9 disruption cycles, respectively (Figure 4).

**Figure 5:**
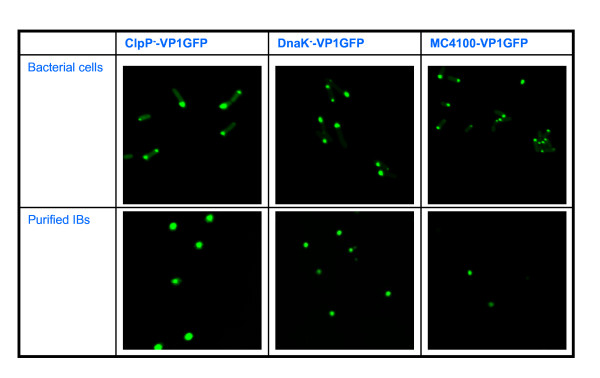
**Confocal microscopy images of ClpP-, DnaK- and MC4100 cells overproducing VP1GFP (top)**. Confocal microscopy images of IBs purified from these strains (bottom).

Since the IB-forming VP1GFP protein, encoded in all the plasmids used here, is a suitable model protein to easily determine functionality, IB architecture and mechanical stability [2,28], we evaluated the degree of IB purity as well as the functionality of the embedded protein, after IB isolation from MC4100/pTVP1GFP, DnaK^-^/pTVP1GFP and ClpP^-^/pTVP1GFP cells, by confocal microscopy. The obtained images confirmed that isolated IBs were still fluorescent and their morphology fully preserved. This demonstrates that the developed protocol does not significantly alter the final protein quality of highly pure IBs (Figure 5).

## Discussion

The presence of bacteria in purified IB samples can be a major drawback when using these nanoparticles for biomedical and industrial applications. Although many IB purification protocols have been developed, their effectiveness regarding residual cell viability had not been tested. In this study we have explored different IB purification methods, focusing our attention on the lysis step, which seems to be decisive to obtain bacteria-free samples. The obtained results clearly show that IB purification methods based on lysozyme treatment, French press or freezing-thawing cycles are not effective concerning complete bacterial cell lysis, while the combination of both sonication and lysozyme treatments was the most effective option (Figure 3). Even though these methods have already been combined in different protocols, cell lysis efficiency remained unproved. Menzella and co-workers and Schrodel and collaborators used 5 min of sonication in order to obtain pure IBs [54,56]. However, our data clearly prove that at least 5 sonication cycles of 10 min at 40% amplitude under 0.5 s cycles are needed to reach a sample completely free from contaminating bacteria. Furthermore, the obtained results indicated that sonication cycles, sonication time but also lysozyme concentration must be determined for each specific protein (Figure 4).

As mentioned before, our results showed a surprising variability among bacterial strains overproducing different recombinant proteins. Villa and collaborators have recently described that membrane lipids are dramatically influenced by the stress resulting from recombinant protein production [59]. Therefore, as the membrane protein composition and permeability in recombinant bacteria can be influenced by the specific produced protein [59,60], the observed variability regarding lysis efficiency could be accounted by different features of the recombinant polypeptide, that dissimilarly causes stress effects on the host cell.

## Conclusion

Results presented here prove that the existing IB purification protocols may be not appropriate when those aggregates have to be used for both catalysis and biomedical purposes, due to residual but significant levels of metabolically active bacterial cells. In this context, a novel protocol developed in this study, which combines both sonication-lysozyme treatment with DNase and detergent washing steps, has proved to be highly efficient regarding cell lysis and useful to obtain preparations of cell-free IBs.

## Materials and methods

### Strains and plasmids

The *Escherichia coli *strains used in this work were MC4100 (*araD139 *Δ(*argF-lac*) *U169 rpsL150 relA1 flbB5301 deoC1 ptsF25 rbsR*, Strep^R^) [61] and their derivatives JGT19 (*clpP::cat *Strep^R^) and JGT20 (*dnak756 thr::Tn10*, Strep^R^, Tc^R^) [62]. The strain MC4100 was transformed with three different plasmids: pTVP1GFP, pTVP1LAC or pTVP1NLSCt encoding engineered versions of GFP and β-galactosidase [7] respectively. The three proteins were fused to the VP1 capsid protein of foot-and-mouth disease virus that dramatically reduces the solubility of the whole fusion, resulting in its aggregation as IBs [22]. JGT10 and JGT20 were only transformed with pTVP1GFP.

### Culture conditions

Bacterial strains were cultured in shake flask cultures at 37°C and 250 rpm in LB rich medium [61] plus 100 μg/ml ampicillin for plasmid maintenance. Recombinant gene expression was induced when the optical density at 550 nm reached 0.5, by adding IPTG to 1 mM. Cell samples were taken at 3 h after induction of gene expression and were processed for bacterial counts, IB sampling and purification and microscopy analyses. Data for further analysis were obtained from three independent experiments.

### Bacterial counts

The concentration of colony forming units (cfu/ml) was determined on LB plates with the corresponding antibiotics. After an appropriate dilution in Ringer 1/4, samples were inoculated on LB plates and incubated at 37°C o/n. Cell counting was always performed in triplicate.

### IBs sampling and purification

Culture samples of 20 ml were taken 3 h after induction and IBs were purified by using two different purification protocols as follows.

*Lysozyme and repeated detergent washing treatment*: cells were harvested by centrifugation at 15,000 g at 4°C for 15 min and resuspended in 400 μl of lysis buffer (50 mM TrisHCl (pH 8.1), 100 mM NaCl and 1 mM EDTA,) and kept at -80°C o/n. After thawing, phenylmethanesulphonylfluoride (PMSF) (2.8 μl, 100 mM) and lysozyme (11.2 μl, 10 mg/ml) were added. After 45 min of incubation at 37°C, 4 μl of Nonidet P40 (NP-40) were added and the mixture incubated at 4°C for 1 h. Then, 12 μl of DNase I (from a 1 mg/ml stock) and 12 μl of 1 M MgSO_4 _were added and the resulting mixture was further incubated at 37°C for 45 min. Protein aggregates were separated by centrifugation at 15,000 g for 15 min at 4°C. Finally, IBs were washed once with 1 ml of the same lysis buffer containing 0.5% Triton X-100. After a final centrifugation at 15,000 g for 15 min at 4°C, pellets were stored at -80°C until analysis. All incubations were done under gentle agitation.

*Lysozyme-detergent, sonication and repeated detergent washing treatment*: samples of bacterial cultures (20 ml) were centrifuged at 4°C at 5,000 g for 5 min and resuspended in lysis buffer (20 ml, 50 mM TrisHCl (pH 8.1), 100 mM NaCl, and 1 mM EDTA) and frozen at -80°C o/n. After thawing, phenylmethanesulphonylfluoride (PMSF) (100 μl, 100 mM) and 1 mg/ml lysozyme (400 μl, 50 mg/ml) were added. After 2 h of incubation at 37°C, 100 μl of Triton X-100 were added (0.5% Triton X-100) and incubated at room temperature for 1 h. Then, the mixture was ice-jacketed, and sonicated between 4 and 10 cycles of 10 min at 40% amplitude under 0.5 s cycles. After sonication, an aliquot of 100 μl of the suspensions were inoculated on LB plates with the corresponding antibiotics and incubated at 37°C o/n. After that, 5 μl of Nonidet P40 (NP-40) were added to the rest of the suspension, and samples incubated at 4°C for 1 h. Then, DNA was removed with DNase (15 μl, 1 mg/ml) and MgSO_4 _(15 μl, 1 M) for 45 min at 37°C. Finally, samples were centrifuged at 4°C at 15,000 g for 15 min, and the pellet containing pure IBs was washed once with 1 ml of lysis buffer containing Triton X-100 (0.5%). After a final centrifugation at 15,000 g for 15 min at 4°C, pellets were stored at -80°C until analysis. All incubations were done under agitation.

### Microscopy analyses of bacteria and IBs

#### Fluorescence microscopy

At 3 h post-induction, VP1GFP-producing cells were fixed with 0.1% formaldehyde in phosphate buffered saline (PBS) and purified IBs were also resuspended in PBS and stored at 4°C until observed. Samples of bacterial cells or IBs were placed on a glass slide, fixed with a slide cover and observed with a Leica TCS SP2 AOBS confocal fluorescence microscope (Leica Microsystems Heidelberg GmbH, Mannheim, Germany) using a Plan-Apochromat objective (zoom 4 or 8; 1024 × 1024 pixels) and optical lens magnification (63×, NA 1.4 oil). Photomicrographs were obtained after excitation at 488 nm and at emission wavelengths between 500 and 600 nm.

#### Scanning Electron Microscopy

Bacterial samples and purified IBs were retained on a nuclepore membrane (Nuclepore Polycarbonate Track-etched Membrane, 0.2 μm pore size, Whatman Ltd.) and fixed with 2.5% phosphate buffered glutaraldehyde (Na_2_HPO_4 _0.9 M, Na_2_H_2_PO_4 _0.06 M, pH 8.0) for 1 h at 4°C. After that, the samples were dehydrated with increasing concentrations of ethanol in water (30, 50, 70, 90 and 100%) by consecutive 5 min washing steps. Ethanol was finally evaporated using the critical point method in a K850 CPD desiccator (Emitech, Ashford, UK). The dried membranes were sputtered with gold using a K550 Sputter Coater (Emitech, Ashford, UK) for observation. Microscopy was performed with a scanning microscope Hitachi S-570 (Hitachi LTD. Tokyo, Japan) using an acceleration between 0.5-30 kV.

## Competing interests

The authors declare that they have no competing interests.

## Authors' contributions

ERC performed most of the experiments and prepared the final data and figures. OCG and JSF purified inclusion bodies and analysed samples by fluorescence microscopy. AV and EGF conceived of the study. EGF directed the work and prepared the manuscript. All authors read and approved the final manuscript.
